# Use of Multiple Adjunctive Negative Pressure Wound Therapy Modalities to Manage Diabetic Lower-Extremity Wounds

**Published:** 2016-12-20

**Authors:** Windy E. Cole

**Affiliations:** Cleveland Clinic Akron General Wound Center, Akron, Ohio

**Keywords:** negative pressure, instillation, hyperbaric oxygen, diabetic foot ulcer, lower extremity

## Abstract

**Objective:** Various treatment options exist for wound healing; however, clinical assessment of the patient and the wound environment must be considered before determining an optimal wound treatment plan. Negative pressure wound therapy alone and/or with an instilled topical solution can be effective in adjunctive management of acute and chronic wounds. Hyperbaric oxygen therapy has also been shown to contribute to the wound-healing process. A pilot evaluation using a multistep approach of adjunctive negative pressure wound therapy with instillation and a dwell time, standard negative pressure wound therapy, and hyperbaric oxygen therapy was explored to manage postsurgical, diabetic lower-extremity wounds with a significant bioburden. **Methods:** Three diabetic patients with lower-extremity ulcers were treated after surgical intervention. Multistep wound therapy consisted of (1) negative pressure wound therapy with instillation of normal saline with a 20-minute dwell time, followed by 2 hours of negative pressure at −150 mm Hg for 3 to 4 days; (2) 1 to 3 weeks of continuous negative pressure at −150 mm Hg; and (3) multiple treatments of hyperbaric oxygen therapy. **Results:** After surgery, wound closure was achieved within 4 weeks postinitiation of multistep wound therapy. All patients regained limb function and recovered with no long-term sequelae. **Conclusions:** In these 3 cases, a multistep wound therapy approach after surgery resulted in successful outcomes; however, larger prospective studies are needed to demonstrate the potential efficacy of this approach in the postsurgical management of complex, diabetic lower-extremity wounds.

The Centers for Disease Control and Prevention estimated the prevalence of diabetes in the United States to be approximately 29.1 million people in 2014.^1^ Diabetic patients are prone to foot ulcer development and have a relatively high risk of infection, gangrene, and amputation.^2-5^ Diabetic lower-extremity wounds often are difficult to heal due to the underlying disease state, patient comorbidities, wound duration, wound location, or a combination of these factors.^6^ Average yearly medical expenditures are about 2.3 times higher for diabetic patients than those for patients without diabetes,^7^ with the annual cost to care for diabetic foot ulcers and lower-extremity amputations of $33,000 and $52,000, respectively.^8^ Diabetic patients with lower-extremity wounds often incur reduced quality of life, with increased workday absenteeism, reduced productivity at work, and increased difficulty in finding or retaining employment.^7^

Patients with diabetes often do not exhibit clinical signs of infection despite high levels of bacteria in chronic wound tissue because peripheral vascular disease, poor metabolic control, and neuropathy diminish first-line inflammatory responses.^9^ Diabetes-related phagocytic and antibacterial dysfunction is thought to be a result of altered glucose metabolism and oxidative stress,^10^ and chronic wounds are often characterized by a high bacterial burden that negatively impacts tissue healing.^11^ Excessive levels of bacterial proteases have been linked to disruptions in the wound-healing process by stimulating a proinflammatory environment and secreting cytokines that promote vasoconstriction and decreased blood flow to the wound bed.^12-14^ Research suggests that some chronic nonhealing wounds may be in a perpetual state of inflammation due to a high bacterial burden.^15-17^ Despite evidence describing the significant challenges to wound healing in the diabetic patient after surgery, an aggressive, multifactorial regimen including negative pressure wound therapy (NPWT), NPWT with instillation and dwell time (NPWTi-d), and hyperbaric oxygen therapy (HBOT) may offer an enhanced approach to managing these complex wounds with a significant bioburden.

Historically, NPWT has not immediately been instituted at our institution when performing incision and drainage or amputation procedures (in instances of abscess and severe infections). Our previous standard protocol included pulsed lavage with normal saline solution (NSS) to thoroughly rinse the wound after surgical intervention, followed by complete postlavage cultures. Wounds would then be packed open for several days awaiting final culture results, and the area would be inspected and monitored for evidence of a continued bioburden.

However, a closed system, such as NPWT or NPWTi-d, could protect the wound from external contamination while also managing bioburden and removing infectious materials during the postsurgical stage.^18,19^ HBOT in addition to NPWT may positively affect granulation tissue and secondary intention healing in cases of severe tissue loss.^20^ We present our experience using a novel multistep wound management strategy of NPWTi-d, NPWT, and HBOT to promote wound healing in diabetic lower-extremity wounds.

## METHODS

All 3 diabetic lower-extremity ulcers in this series contained large amounts of devitalized tissue that required surgical intervention. Osteomyelitis was diagnosed in each wound, leading to resection of the affected bone and was treated before starting any therapy. Surgical intervention consisting of incision and drainage of the abscess with amputation or removal of the nonviable tissues was performed in all cases. Once all nonviable tissue was surgically excised, a pulsed lavage system with NSS was used to copiously flush the wound. Postlavage cultures were then obtained to administer the appropriate antibiotic therapy. NPWTi-d (V.A.C. VERAFLO Therapy; KCI, an ACELITY Company, San Antonio, Tex) was applied in the operating room (OR) immediately following the surgical procedure. NSS was instilled into the wound with a 20-minute dwell time, followed by 2 hours of negative pressure at −150 mm Hg. Dressings were changed every other day. Following sufficient wound-healing progress, patients were discharged home on NPWT (V.A.C. Therapy; KCI, an ACELITY Company). Patients also received multiple 90-minute HBOT sessions of 100% oxygen at 2.4 atm in the outpatient setting. If HBOT was started while on NPWT, manufacturer guidelines were followed and the patient was disconnected from the NPWT unit before entering the HBOT chamber.

## RESULTS

Three male patients (average age 53.0 years) with diabetic lower-extremity ulcers were managed with multistep wound therapy after surgical intervention. Wound closure was observed within 4 weeks postinitiation of multistep wound therapy. All patients regained limb function and recovered with no reinfections or additional complications. The 3 cases are detailed further to illustrate the use of multistep wound therapy.

### Case 1: Diabetic foot ulcer

**Figure 1:**
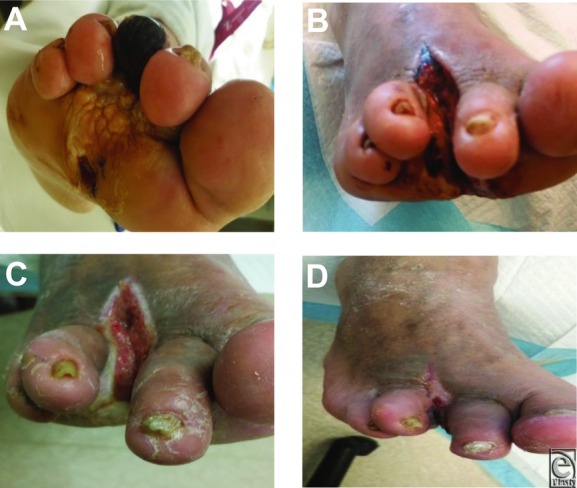
(a) Wound at initial presentation. (b) Postoperative day 2, wound appearance after amputation and 2 days of NPWTi-d with normal saline. (c) Postoperative day 4, wound appearance at the time of hospital discharge. (d) Postoperative day 10, wound appearance after 1 week of traditional NPWT and HBOT.

A 43-year-old diabetic man, with a medical history of hyperlipidemia and hypertension, presented with peripheral neuropathy with a deep probing ulcer on the sub-third metatarsal head that was gangrenous. Exudate was foul and purulent, and there was crepitation of the surrounding soft-tissue structures (Fig 1*a*). The patient was admitted into the hospital for wide excision and amputation of the third ray and two thirds of the third metatarsal. Immediately following the partial ray amputation, NPWTi-d was initiated. After 2 days of NPWTi-d, the base of the wound was beefy red and granular. It had filled in considerably, and no evidence of contamination or devitalized tissue was noted (Fig 1*b*). On postoperative day 4, the wound was well granulated with epithelializing wound edges (Fig 1*c*) and NPWTi-d was discontinued. The patient was discharged home on NPWT and received HBOT in the outpatient setting. NPWT was discontinued after 1 week, as the wound displayed complete granulation with no evidence of contamination, odor, or other complications (Fig 1*d*). Following multiple treatments of HBOT, an acellular dermal matrix (DermACELL, Novadaq Technologies Inc, Mississauga, Ontario, Canada) was applied to the wound. The wound completely healed, and the patient had restored limb function.

### Case 2: Foot abscess and cellulitis

**Figure 2:**
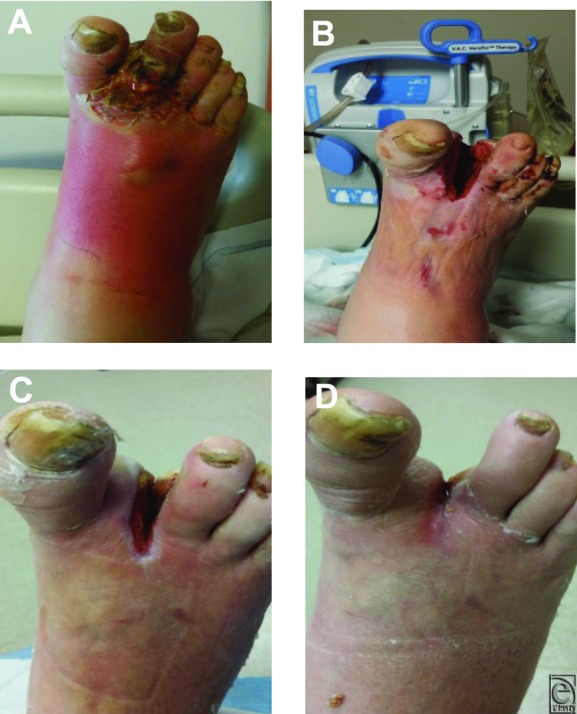
(a) Wound at initial presentation. (b) Postoperative day 2, wound appearance after amputation and 2 days of NPWTi-d with normal saline. (c) Postoperative day 10, wound appearance after 1 week of NPWT. (d) Postoperative day 29, wound appearance after 3 weeks of NPWT and HBOT.

A 52-year-old man with insulin-dependent diabetes mellitus presented to the wound care clinic with a 3-day history of abscess to the right foot (Fig 2*a*). Upon evaluation, cellulitis of the right foot was noted to the level of the ankle joint. There was marked warmth to the entire foot with fluctuance of the tissues in the midfoot and appreciable abscess accumulation subdermally. Foul, purulent discharge was actively draining from the ulcerated area of the second digit. At the time of hospital admission, the patient's diabetes was uncontrolled with a glucose reading of 350 mg/dL, with complaints of peripheral neuropathy. A magnetic resonance image showed osteomyelitis of the toe and second metatarsal, and the patient was taken to the OR where second toe amputation and ray resection were performed. NPWTi-d was initiated immediately postamputation in the OR. After 2 days of NPWTi-d, erythema and edema were dramatically reduced (Fig 2*b*). The base of the wound was beefy red and granular without signs of devitalized tissue or slough. On postoperative day 4, NPWTi-d was discontinued and the patient was discharged home on NPWT. After 1 week of NPWT, the wound displayed increased granulation tissue and reepithelialization (Fig 2*c*). After 3 weeks, NPWT was discontinued (Fig 2*d*) and HBOT was started. The wound continued to display increased granulation tissue and decreased wound depth area, with complete closure within 4 weeks postinitiation of therapy.

### Case 3: Left ankle abscess

**Figure 3:**
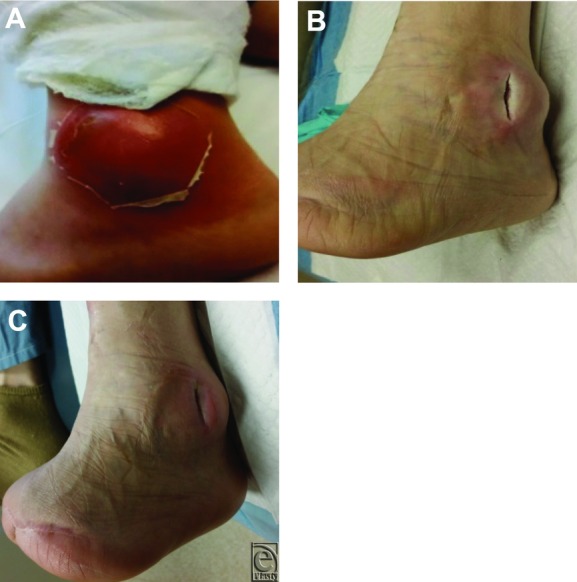
(a) Wound at initial presentation. (b) Postoperative day 2, wound appearance after NPWTi-d with normal saline. (c) Postoperative day 20, wound appearance after 3 weeks of NPWT and HBOT.

A 64-year-old man, with insulin-dependent diabetes mellitus and a medical history of transmetatarsal amputation of the left foot due to gangrene and osteomyelitis, presented with a 3-day history of erythema and pronounced edema in the area of the left lateral ankle (Fig 3*a*). At the time of presentation, he was completely neuropathic with advanced cellulitis and fluctuance with palpation. Magnetic resonance imaging finding led to the diagnosis of osteomyelitis. The patient was taken to the OR where surgical excision of the osteomyelitic bone was performed along with incision and drainage of the abscess. A bone culture revealed methicillin-resistant *Staphylococcus aureus*, and the patient was started on intravenous vancomycin (1 g every 12 hours). Immediately following surgery, NPWTi-d was initiated. On postoperative day 2, the wound base was beefy red and granular, and the erythema and edema had resolved (Fig 3*b*). On postoperative day 3, NPWTi-d was discontinued and the patient was discharged home on NPWT, followed by HBOT. After 2 weeks of NPWT and HBOT, the wound displayed only a superficial fissure at the site where the previous irrigation and drainage were performed (Fig 3*c*). After 4 weeks of multistep therapy, wound closure was observed.

## DISCUSSION

Poor healing and/or infection-related complications often delay and compromise treatment of diabetic lower-extremity wounds. However, we managed these complex diabetic foot ulcers with multistep wound therapy, which resulted in wound closure within 4 weeks of postsurgical application.

Various diabetic lower-extremity wound treatments have been reported in the literature, including NPWTi-d,^14,19^ NPWT,^3,21,22^ and HBOT,^23-25^ and 2 previous studies have also supported the combined use of NPWT and HBOT in the management of necrotizing fasciitis of the breast^26^ and limb-threatening, ischemic, diabetic foot ulcers.^27^ Thus, individually, these therapies can promote wound healing in complex wounds through their unique mechanisms of action. NPWT has been shown to promote perfusion, reduce edema, remove exudate and infectious materials from the wound bed, promote granulation tissue formation, and draw wound edges together.^21,28-30^ Furthermore, allowing an instilled topical solution to dwell in the wound during NPWTi-d may assist in cleansing the wound as well as diluting and solubilizing infectious materials, followed by removal via the negative pressure phase.^18^ Indeed, intermittent instillation of 0.9% normal saline during NPWTi-d has shown positive clinical outcomes.^19,31,32^ Furthermore, HBOT with routine surgical and antibiotic management has been demonstrated to be an effective adjunctive wound therapy for osteomyelitis,^33^ and exposure to this treatment has led to increased neovascularization and fibroblast proliferation in cases of ischemia and infection.^34,35^ HBOT also has been shown to potentiate the activity of certain antibiotics by decreasing bacterial viability and increasing polymorphonuclear leukocyte activity.^36,37^

All the patients in this pilot evaluation required aggressive surgical intervention, which resulted in deep tissue loss. Unfortunately, our previous wound therapy protocol resulted in poor clinical outcomes or slow rates of wound improvement. However, recognizing the benefits of each therapy and utilizing a multistep therapy approach allowed for earlier use of NPWTi-d, NPWT, and HBOT in the treatment plan. Compared with previous postsurgical regimens, starting multistep wound therapy sooner resulted in improved healing and successful outcomes for these 3 patients. There were no complications or reinfections during the course of wound management. All patients in this pilot evaluation regained limb function, and they were able to make a complete recovery with no long-term sequelae. In light of the successful outcomes of these 3 patients, larger prospective studies evaluating multistep wound therapy on wound bioburden and diabetic lower-extremity outcomes are needed to design definitive treatments.
